# Genome Sequence of *Corynebacterium pseudotuberculosis* MB20 bv. equi Isolated from a Pectoral Abscess of an Oldenburg Horse in California

**DOI:** 10.1128/genomeA.00977-14

**Published:** 2014-11-13

**Authors:** Rafael A. Baraúna, Luís C. Guimarães, Adonney A. O. Veras, Pablo H. C. G. de Sá, Diego A. Graças, Kenny C. Pinheiro, Andreia S. S. Silva, Edson L. Folador, Leandro J. Benevides, Marcus V. C. Viana, Adriana R. Carneiro, Maria P. C. Schneider, Sharon J. Spier, Judy M. Edman, Rommel T. J. Ramos, Vasco Azevedo, Artur Silva

**Affiliations:** a Institute of Biological Sciences, Federal University of Pará, Belém, PA, Brazil; b Institute of Biological Sciences, Federal University of Minas Gerais, Belo Horizonte, MG, Brazil; c Department of Medicine and Epidemiology, School of Veterinary Medicine, University of California Davis, California, USA

## Abstract

The genome of *Corynebacterium pseudotuberculosis* MB20 bv. equi was sequenced using the Ion Personal Genome Machine (PGM) platform, and showed a size of 2,363,089 bp, with 2,365 coding sequences and a GC content of 52.1%. These results will serve as a basis for further studies on the pathogenicity of *C. pseudotuberculosis* bv. equi.

## GENOME ANNOUNCEMENT

Recent advances have been made in the genomic analysis of *Corynebacterium pseudotuberculosis*, a species of veterinary and biotechnological interest. Molecular diagnosis of several diseases caused by this species was achieved using multiplex PCR (PCR) (1), and a description of its pangenome was published using 15 strains of *C. pseudotuberculosis* bv. ovis and equi (2). *C. pseudotuberculosis* bv. equi comprises strains that infect horses and cattle and cause infection of cutaneous lymphatic vessels, termed ulcerative lymphangitis, which is characterized by the development of multiple waxy ulcerative lesions. The incidence of this disease in horses has been reported in the literature since the 1910s (3) and remains prevalent in animals worldwide (4, 5); moreover, this infection is likely underreported and has been characterized as a neglected zoonosis (6).

The host-pathogen interaction in this disease has been studied using omics approaches (7). For instance, the differential gene expression of a *C. pseudotuberculosis* bv. ovis strain was analyzed using RNA-seq, and genes involved in the molecular responses of the bacterium to different stresses during infection were identified (8). A study of reverse vaccinology reported by Soares et al. (9) identified 49 possible antigens from the genome of the *C. pseudotuberculosis* bv. equi strain 258 that may serve as targets for the development of effective vaccines. In addition, a new *C. pseudotuberculosis* bv. equi strain was isolated and sequenced, which will aid in future broader studies. These new data combined with those already reported will serve as a basis for the development of studies aimed at a better understanding of the pathogenic potential of *C. pseudotuberculosis* bv. equi.

The MB20 strain was isolated from a pectoral abscess of a 4-year-old horse of the breed Oldenburg, raised in the city of Vacaville, CA, USA. Genomic DNA was sequenced from a fragment library on a 318 chip of the Ion Torrent Personal Genome Machine (PGM) platform (Life Technologies). A total of 2,331,864 reads were generated with an average length of 420 bp, which were used for genome assembly using the software Mira (10). The contigs generated with Mira were analyzed using the SeqMan Pro tool of the software Lasergene 11 Core Suite (DNASTAR) to remove redundant sequences. This approach resulted in 3 contigs, which were sorted with the Artemis Comparison tool (11) using the genome of *C. pseudotuberculosis* 316 as a reference. The scaffold produced at the end of the assembly was 2,363,089 bp in size and underwent automatic annotation using Rapid Annotation using Subsystem Technology (RAST) (12). As a result, 2,365 coding sequences (CDSs), 11 rRNA genes, 51 tRNA genes and a 52.1% GC content were identified. Of the 2,365 CDSs, 790 (33.4%) were classified as hypothetical proteins.

### Nucleotide sequence accession numbers.

The genomic sequence obtained in this study was deposited in the DDBJ/EMBL/GenBank under accession number JPUV00000000. The version described in this paper is version JPUV01000000.

## References

[1] Pacheco LGC Pena RR Castro TLP Dorella FA Bahia RC Carminati R Frota MNL Oliveira SC Meyer R Alves FSF Miyoshi A Azevedo V 2007 Multiplex PCR assay for the identification of *Corynebacterium pseudotuberculosis* from pure cultures and for rapid detection of this pathogen in clinical samples. J. Med. Microbiol. 56:480–486. 10.1099/jmm.0.46997-0. 17374887

[2] Soares SC Silva A Trost E Blom J Ramos R Carneiro A Ali A Santos AR Pinto AC Diniz C Barbosa EG Dorella FA Aburjaile F Rocha FS Nascimento KK Guimarães LC Almeida S Hassan SS Bakhtiar SM Pereira UP Abreu VA Schneider MP Miyoshi A Tauch A Azevedo V 2013 The pan-genome of the animal pathogen *Corynebacterium pseudotuberculosis* reveals differences in genome plasticity between the biovar ovis and equi strains. PLoS One 8:e53818. 10.1371/journal.pone.0053818. 23342011 PMC3544762

[3] Hall IC Stone RV 1916 The diphtheroid bacillus of Preisz-Nocard from equine, bovine, and ovine abscesses: ulcerative lymphangitis and caseous lymphadenitis. J. Infect. Dis. 18:195–208. 10.1093/infdis/18.2.195.

[4] Hepworth-Warren KL Sponseller BT Wong DM Kinyon JM 2014 Isolation of *Corynebacterium pseudotuberculosis* biovar equi from a horse in central Iowa. Case Rep. Vet. Med. 2014:1–3. 10.1155/2014/436287.

[5] Spier SJ Leutenegger CM Carroll SP Loye JE Pusterla JB Carpenter TE Mihalyi JE Madigan JE 2004 Use of a real-time polymerase chain reaction-based fluorogenic 5′ nuclease assay to evaluate insect vectors of *Corynebacterium pseudotuberculosis* infections in horses. Am. J. Vet. Res. 65:829–834. 10.2460/ajvr.2004.65.829. 15198224

[6] Join-Lambert OF Ouache M Canioni D Beretti JL Blanche S Berche P Kayal S 2006 *Corynebacterium pseudotuberculosis* necrotizing lymphadenitis in a twelve-year-old patient. Pediatr. Infect. Dis. J. 25:848–851. 16940849 10.1097/01.inf.0000234071.93044.77

[7] Dorella FA Gala-Garcia A Pinto AC Sarrouh B Antunes CA Ribeiro D Aburjaile FF Fiaux KK Guimarães LC Seyffert N El-Aouar R Silva R Hassan SS Castro TLP Marques WS Ramos R Carneiro A Sá P Miyoshi A Azevedo V Silva A 2013 Progression of “OMICS” methodologies for understanding the pathogenicity of *Corynebacterium pseudotuberculosis*: the Brazilian experience. Comput. Struct. Biotechnol. J. 6 e201303013. 10.5936/csbj.201303013. 24688721 PMC3962224

[8] Pinto AC Sá PH Ramos RT Barbosa S Barbosa HP Ribeiro AC Silva WM Rocha FS Santana MP Castro TL Miyoshi A Schneider MP Silva A Azevedo V 2014 Differential transcriptional profile of *Corynebacterium pseudotuberculosis* in response to abiotic stresses. BMC Genomics 15:14. 10.1186/1471-2164-15-14. 24405787 PMC3890534

[9] Soares SC Trost E Ramos RT Carneiro AR Santos AR Pinto AC Barbosa E Aburjaile F Ali A Diniz CA Hassan SS Fiaux K Guimarães LC Bakhtiar SM Pereira U Almeida SS Abreu VA Rocha FS Dorella FA Miyoshi A Silva A Azevedo V Tauch A 2013 Genome sequence of *Corynebacterium pseudotuberculosis* biovar equi strain 258 and prediction of antigenic targets to improve biotechnological vaccine production. J. Biotechnol. 167:135–141. 10.1016/j.jbiotec.2012.11.003. 23201561

[10] Chevreux B Pfisterer Drescher B Driesel AJ Müller WEG Wetter T Suhai S 2004 Using the miraEST assembler for reliable and automated mRNA transcript assembly and SNP detection in sequenced ESTs. Genome Res. 14:1147–1159. 10.1101/gr.1917404. 15140833 PMC419793

[11] Carver TJ Rutherford KM Berriman M Rajandream MA Barrell BG Parkhill J 2005 ACT: the Artemis comparison Tool. Bioinformatics 21:3422–3423. 10.1093/bioinformatics/bti553. 15976072

[12] Overbeek R Olson R Pusch GD Olsen GJ Davis JJ Disz T Edwards RA Gerdes S Parrello B Shukla M Vonstein V Wattam AR Xia F Stevens R 2014 The SEED and the Rapid Annotation of microbial genomes using Subsystems Technology (RAST). Nucleic Acids Res. 42:D206–D214. 10.1093/nar/gkt1226. 24293654 PMC3965101

